# Correction: Study of near-infrared light-induced excitation of upconversion nanoparticles as a vector for non-viral DNA delivery

**DOI:** 10.1039/d0ra90128h

**Published:** 2021-01-04

**Authors:** Jen-Hsuan Wang, Hsin-Yu Chen, Ching-Cheng Chuang, Jung-Chih Chen

**Affiliations:** Institute of Biomedical Engineering, National Chiao Tung University HsinChu 30010 Taiwan Republic of China george@nctu.edu.tw; Department of Electrical and Computer Engineering, National Chiao Tung University HsinChu 30010 Taiwan Republic of China; Department of Biological Science and Technology, National Chiao Tung University HsinChu 30010 Taiwan Republic of China

## Abstract

Correction for ‘Study of near-infrared light-induced excitation of upconversion nanoparticles as a vector for non-viral DNA delivery’ by Jen-Hsuan Wang *et al.*, *RSC Adv.*, 2020, **10**, 41013–41021, DOI: 10.1039/D0RA05385F.

The authors regret that incorrect versions of Fig. 6 and 7 were included in the original article. The correct versions of Fig. 6 and 7 are presented below.

**Fig. 6:**
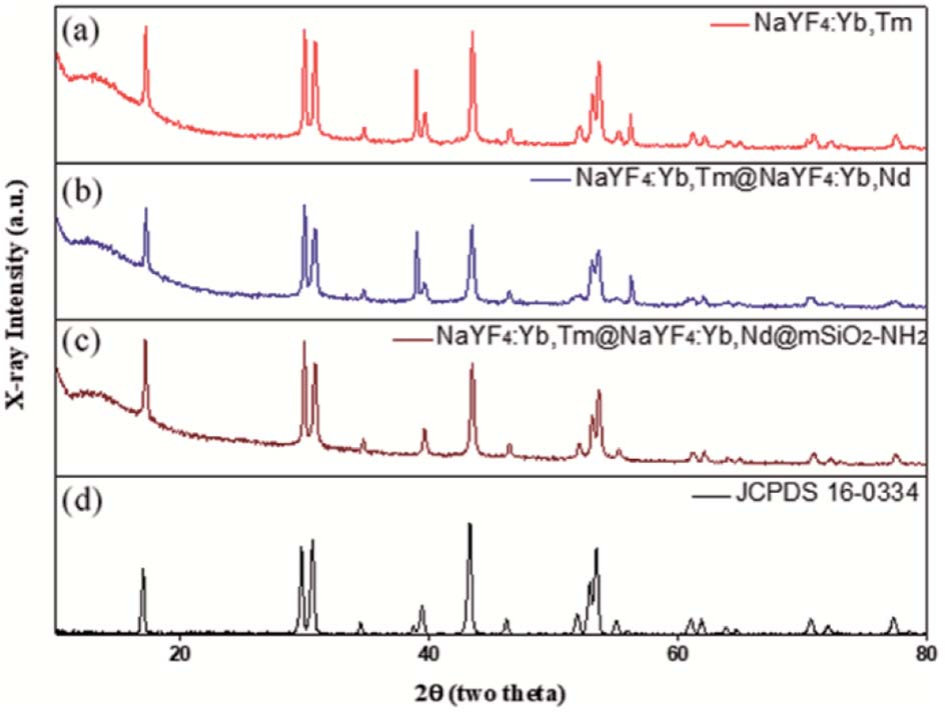
XRD patterns of (a) NaYF_4_:Yb,Tm, (b) NaYF_4_:Yb,Tm@NaYF_4_:Yb,Nd, (c) NaYF_4_:Yb,Tm@NaYF_4_:Yb,Nd@mSiO_2_-NH_2_, and (d) hexagonal phase (JCPDS standard card no. 16-0334).

**Fig. 7:**
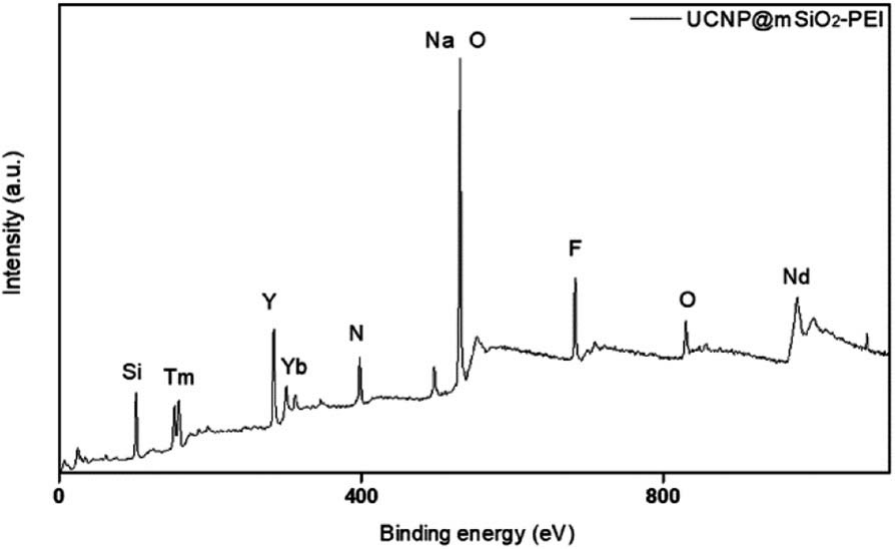
XPS pattern of UCNP@mSiO_2_-PEI.

The Royal Society of Chemistry apologises for these errors and any consequent inconvenience to authors and readers.

## Supplementary Material

